# Manual of Diseases and Deformities of the Spine

**Published:** 1895-06

**Authors:** 


*Manual of Diseases and Deformities of the Spine.* By R. L. SWAN. Pp. xvi., 194. Dublin: FANNIN & CO. 1894.

This is evidently the work of a surgeon who has had large experience in the treatment of spinal diseases and deformities, for every page bears evidence of good sound knowledge. The important subjects of angular and lateral curvatures of the spine are well and fully dealt with, and the methods of treatment recommended are eminently practical and good. We notice that the author speaks with high approval of Sayre's plaster-of-Paris jackets in spinal caries, and that he prefers the horizontal suspension on a hammock to the vertical method of suspension by the head and arms. We are quite of his opinion, but we do not see why he should always speak of Sayre as "Sayer."

In the treatment of paraplegia—the result of caries—the operation of laminectomy is only slightly mentioned, and not described at all, while under the head of fractures and dislocations of the spine the operation is not even alluded to; so we suppose it does not meet with the author's approval. The chapters on Spina Bifida, Torticollis, and other congenital spinal affections are good; but it is a pity the author thought fit to illustrate (?) the book with drawings which we can only call disfigurements, while if he had prevailed upon some literary friend to look over the proof-sheets they would not have contained upon almost every page the innumerable errors which we now find.

